# Magnetic and Magneto-Optical Oroperties of Iron Oxides Nanoparticles Synthesized under Atmospheric Pressure

**DOI:** 10.3390/nano10091888

**Published:** 2020-09-21

**Authors:** Aleksandr Spivakov, Chun-Rong Lin, Yu-Chuan Chang, Cheng-Chien Wang, Dmitriy Sarychev

**Affiliations:** 1 Department of Applied Physics, National Pingtung University, Pingtung County 90003, Taiwan; aleksandr.a.spivakov@gmail.com (A.S.); xjp004114.ni0729@gmail.com (Y.-C.C.); 2 Research Institute of Physics, Southern Federal University, Rostov-on-Don 344090, Russia; 3 Department of Chemical and Materials Engineering, Southern Taiwan University of Science and Technology, Tainan city 710, Taiwan; ccwang@stust.edu.tw

**Keywords:** magnetite nanoparticles, MCD spectroscopy, size effect, thermal decomposition, synthesis conditions

## Abstract

Magnetite nanoparticles were synthesized by a simple thermal decomposition process, involving only iron (III) nitrate nonahydrate as a precursor, and hexadecylamine as a solvent and stabilizer at reaction temperatures varied from 200 to 380 °C. The results of the structural analysis showed that the average crystallite size depends on the reaction temperature and increases from 4.8 to 13.3 nm. The behavior of the coercivity indicates that all synthesized samples are single domain; herewith, it was found that the critical size corresponding to the transition to the superparamagnetic state at room temperature is about 9 nm. The effect of the reaction temperature on changes in the saturation magnetization was studied. It was found that the size effect in the MCD spectra is observed for the IVCT transition and one ISCT transition, and the influence of the reaction temperature on the change in the MCD spectra was discussed.

## 1. Introduction

Over the past decade, there has been an increasing interest in iron oxide nanoparticles (IONs), caused not only by fundamental scientific interest but also by their practical application. Due to the unique size-dependent magnetic properties, IONs are used in a wide variety of fields, including nanocatalysis, all-optical switching devices, microwave absorption, environmental remediation, chemical and biological sensors, energy generation and storage, biomedicine and biotechnologies, etc. [1,2,3,4,5,6,7,8,9,10,11,12]. Among various IONs, magnetite (Fe_3_O_4_) nanoparticles are one of the most promising, since they find application in such areas as high-density magnetic storage media, capacitor electrodes, development of sensors, ferrofluid, for absorption heavy metals [5,13,14,15,16,17,18,19], have wide application in biotechnologies and biomedicine, for example, magnetic resonance imaging, hyperthermia, targeted delivery of drugs, detect and treat cancer [20,21,22,23,24,25,26].

Many different methods for the synthesis of magnetite nanoparticles have been described in the literature, including co-precipitation [27,28,29], hydrothermal [30,31,32], sol-gel [33,34,35], thermal decomposition [36,37,38,39,40], among others [41,42,43]. Besides, the key role of the synthesis conditions, such as reaction temperature, reagents concentration, the effect of different additives, and others on the magnetic properties and size control have also been discussed in the literature. Such methods as sol-gel and hydrothermal have a long reaction time and/or occur at high pressure [44,45], which reduces the possibility of their widespread use. Among these techniques, the method of thermal decomposition (TD) of various iron complexes using solvents with high boiling points has demonstrated promising results for obtaining of size-controlled, superparamagnetic magnetite nanoparticles. The most common iron complex for the TD method is iron (III) acetylacetonate (Fe(acac)_3_) [39,46,47]; in addition, the use of other precursors [37,48,49] has also been described in the literature. However, most of the thermal decomposition processes use expensive reagent mixtures, for example, with 1,2-hexadecanediol or they proceed under complicated conditions. All this complicates the development of magnetite nanoparticles and their study for practical application. Therefore, the development of simple synthesis methods without the use of expensive reagents and the study of the influence of synthesis conditions on the physicochemical properties of magnetite nanoparticles is an important task contributing to the progress of their practical application.

In this regard, the use of other solvents and iron complexes is of particular interest and has been investigated in the literature [36,37,39,47]. Therefore, hexadecylamine (HDA) has been used as a solvent, stabilizing and reducing agent for the synthesis of nanoparticles of silver [50], nickel [51], bismuth [52], and others. Authors of work [53] have synthesized superparamagnetic magnetite nanoparticles via the solvothermal method by the reduction of Fe(acac)_3_ using a mixture with HDA as a stabilizer. However, the uses of other synthesis methods and precursors, as well as the influence of synthesis conditions on the formation of magnetite nanoparticles, have not been described in the literature.

In this work, we present a simple and low-cost method for the synthesis of magnetite nanoparticles by thermal decomposition process involving only two chemicals: iron (III) nitrate nonahydrate (Fe(NO_3_)_3_·9H_2_O) and HDA, which was used as a solvent and stabilizer. As far as we know, the thermal decomposition process of Fe(NO_3_)_3_·9H_2_O using HDA has not been described previously. Besides, the synthesis process proceeds without the use of protective gas (such as nitrogen or argon) and at atmospheric pressure, making it easily reproducible. The influence of the reaction temperature on the phase purity, magnetic and magneto-optical properties of magnetite nanoparticles has been investigated.

## 2. Materials and Methods

### 2.1. Synthesis of Magnetite Nanoparticles

Magnetite nanoparticles were synthesized by a thermal pyrolysis method, using HDA as a stabilizing agent. Analytically pure reagents were used as the starting materials without further purification. In a typical synthesis process, 12.2 g of 1–hexadecylamine (Fisher Scientific International, Inc., Pittsburgh, PA, USA) was melted at 80 °C in a round-bottom flask, and then 2.9 g of iron (III) nitrate nonahydrate (Merck Millipore, Burlington, MA, USA) (Fe(NO_3_)_3_·9H_2_O) was dissolved in it. To remove water from iron nitrate, the mixture was heated to 120 °C for 30 min under magnetic stirring. To obtain nanoparticles with different sizes, the mixture was heated to the final temperature of the reaction (T_R_), which was varied from 200 to 380 °C and held at appropriate temperatures for 1 h. After completion of the reaction, the resulting black solution was cooled to 70 °C and washed several times with toluene, which ensures complete removal of HDA from the samples.

### 2.2. Characterizations

The crystal structure and morphology of the nanoparticles were characterized by X-ray diffraction (XRD) measurements using a Bruker D8 Advance diffractometer (Bruker Corp., Billerica, MA, USA) (Cu Kα radiation, 40 kV, 25 mA, λ = 1.5418 Å) and transmission electron microscopy (TEM) (JEOL JEM-1230 (JEOL Ltd., Japan, Tokyo) microscope operated at an accelerating voltage of 80 kV). The Raman spectra were obtained using a Shamrock 750 spectrograph (Andor Technology Ltd., Belfast, Northern Ireland) equipped with a CCD detector. The 533-nm line from the CW He–Ne randomly polarized laser was used for excitation at 0.45 mW laser power. Magnetic properties, such as the saturation magnetization (M_s_) and coercivity (H_c_) were studied via a vibrating sample magnetometer (Lakeshore 7400 series VSM (Lake Shore Cryotronics Inc., Westerville, OH, USA)) in the applied field of H = ±15 kOe. Magnetic circular dichroism (MCD) spectra were recorded at room temperature in a magnetic field of 8 kOe with a JASCO J-820 spectropolarimeter (JASCO Inc., Mary’s CourtEaston, MD, USA) equipped with an electromagnet GMW Associates 5403. All measurements were carried out at ambient conditions.

## 3. Results and Discussions

### 3.1. Structural Characterization

**Figure 1:**
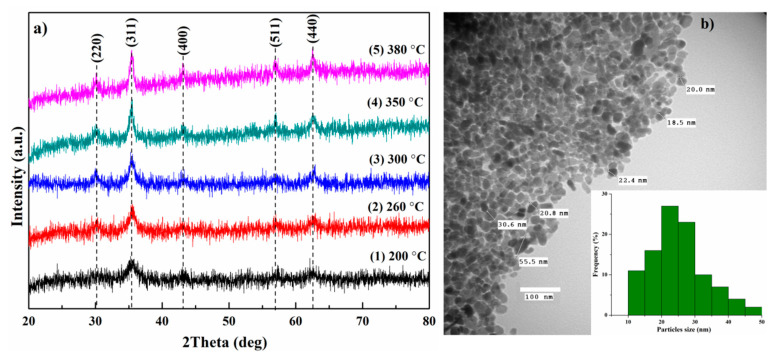
(**a**) XRD patterns of the samples synthesized at different reaction temperatures and (**b**) TEM micrograph of magnetite nanoparticles synthesized at 380 °C. The inset shows the histogram of particle size distribution.

The XRD patterns of the samples synthesized at different reaction temperatures are shown in Figure 1. It can be seen from the figure that with an increase in the reaction temperature, the peaks become narrower and sharper, which indicates an increase in crystallite size and better crystallinity. The reflections in the patterns are consistent with the cubic inverse spinel structure of magnetite (JCPDS 19-0629) [54] with space group $Fd\bar{3}m$ and do not contain features of other oxides such as hematite, goethite or wustite. However, since magnetite and maghemite (γ-Fe_2_O_3_) have similar XRD patterns with close reflections, they are difficult to be distinguished by XRD. Therefore, to better distinguish the phases of magnetite and maghemite, Raman spectroscopy was used further.

The average crystallite sizes were calculated using Scherrer’s equation (1) [55] from the broadening of the most intense peak (311) and the results are listed in Table 1.

(1) $$d_{XRD}=\frac{0.89\lambda }{\beta cos\theta }$$

where *λ* is the radiation wavelength (0.5418 nm for Cu K*α*); *β* is the line broadening of a diffraction peak at angle *θ*.

The results show that an increase in the reaction temperature leads to a change in crystallite size from 4.8 to 13.3 nm. Thus, in this synthesis process, crystallite size control can be easily carried out by changing the reaction temperature.

The lattice parameter of the samples was calculated based on Bragg’s law by the following relation and obtained results are also presented in Table 1.

(2) $$a=d_{hkl}\sqrt{h^{2}+k^{2}+l^{2}}$$

where *d_hkl_*—inter planar distance; (hkl) is the Miller indices of the planes.

The results show that the lattice constant is slightly smaller than that of bulk magnetite (a_bulk_ ≈ 8.4) [54]. The difference between these values can be explained by the presence of a small fraction of maghemite in the samples (a_magh._ = 8.35 [56]). Therein, with an increase in the reaction temperature, the values of the lattice parameter shift toward higher values, which may indicate a decrease in the fraction of γ-Fe_2_O_3_ in the samples. However, Lemine et al. [33] have synthesized pure magnetite nanoparticles, which was confirmed by Mössbauer spectroscopy, and have obtained the value of lattice parameter *a* = 8.3639 for nanoparticles with a size of 8 nm. This result has been explained by the size effect on the crystal lattice. Both these factors probably influence the change in the lattice parameter with the increase in the reaction temperature, and, as a consequence, with a change in the size of nanoparticles.

The TEM image of the sample synthesized at 380 °C is shown in Figure 1b and the inset demonstrates particle size distribution. It can be seen that nanoparticles have a quasi-spherical shape with a tendency to agglomerate. The particle size of the sample oscillates between 10 and 50 nm (insert in Figure 1b), with an average size of 24 ± 2 nm. This value is larger than calculated from the XRD data and the same tendency is observed for all samples. The difference between size obtained by TEM and XRD can be explained by Ostwald ripening process, wherein small nanoparticles dissolved in supersaturated solution and become larger since equilibrium of the solution is unstable [57]. In addition, a reasonable agreement of the obtained values indicates a balance between stabilization and crystal growth in HDA.

**Figure 2:**
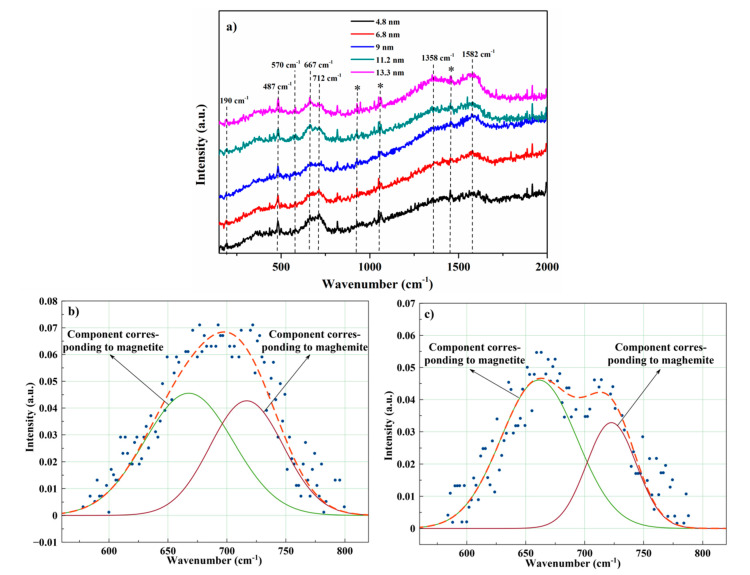
Raman spectra of magnetite nanoparticles synthesized at different reaction temperatures (**a**) and the best Gaussian fitting for the A_1g_ peaks of the samples with d = 4.8 nm (**b**) and 13.3 nm (**c**); *—artifacts.

To better distinguish the phases of iron oxides in the samples, Raman spectroscopy was performed. Under normal conditions, magnetite has the space group $Fd\bar{3}m$ and group theory analysis by White and DeAngelis [58] predicts five Raman active modes: A_1g_ + E_g_ + 3T_2g_. The Raman spectra of the samples in the range of 150–2000 cm^−1^ are presented in Figure 2 and the identification of Raman modes was carried out based on literature data obtained for various iron oxides.

**Figure 3:**
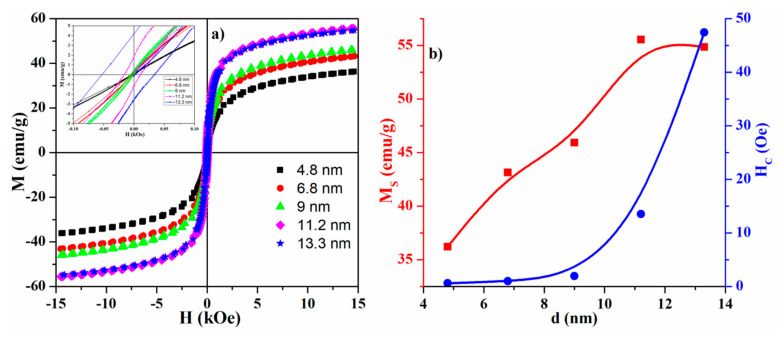
(**a**) Magnetic hysteresis loops of the magnetite nanoparticles; (**b**) size dependence of saturation magnetization and coercivity. The inset shows the hysteresis loops in an enlarged scale.

The Raman peak at ~667 cm^−1^ is assigned to A_1g_ mode and corresponds to the symmetrical Fe-O stretch [59,60,61] and is a clear indication of the magnetite phase. The peaks observed at 190 and 570 cm^−1^, which is more clearly observed with an increase of particle size, confirm the phase of magnetite and are assigned to T_2g_(1) and T_2g_(3) modes [62,63], respectively. The position of the peak at 487 cm^−1^ corresponds to the T_2g_(2) mode of magnetite [64,65]; however, this peak should be weaker than that shown in Figure 3. We assume that the peak at 487 cm^−1^ is a mix of the T_2g_(2) mode of magnetite and E_g_ mode of maghemite. The band at about 712 cm^−1^ is the sign of the A_1g_ mode of maghemite [64,66], which may be due to the oxidation of magnetite to maghemite, which occurs more actively at low laser intensity [64], as in the case of our experiment. Further, for the samples synthesized at high temperatures, the peak at 712 cm^−1^ is separated from the peak of magnetite and has a lower intensity. This fact, along with an increase in peak intensity at 570 cm^−1^, allows us to conclude that an increase in the reaction temperature leads to the formation of more phase-pure samples. We have semi-quantitatively estimated the ratio magnetite/maghemite based on the results of Raman spectroscopy. Figure 2b,c show the decomposition of the A_1g_ peak for the samples synthesized at 200 and 380 °C into two Gaussian components, which correspond to the contributions of magnetite and maghemite to the peak. The fitting results showed that the contribution of the components (magnetite/maghemite) to the peak varies from ~54.83%/45.17% (for the sample with size d_XRD_ = 4.8 nm) to 60.33%/39.67% (for the sample with size d_XRD_ = 13.3 nm). Dubois and co-authors [67] have determined a correlation between the contribution of the magnetite and maghemite to the experimental Raman spectra and the weight fraction of each oxide in the samples. Based on the results obtained in work [67], we calculated the ratio magnetite/maghemite in the synthesized samples. This semi-quantitative estimate showed that the ratio is ~93%/7% (for the sample with d_XRD_ = 4.8 nm) and ~94%/6% (for the sample with d_XRD_ = 13.3 nm. The Raman peaks at about 1358 and 1582 cm^−1^ are in a good agreement with the positions of D and G bands of carbon. The D peak is associated with defects in the hexagonal *sp*^2^ C network or the finite particle size effect, whereas the G peak arises from the stretching of the C–C bond in graphitic materials, and is common to all *sp*^2^ carbon systems [68]. The presence of carbon in the samples can be explained by the synthesis process using HDA, during which carbon was formed. As a result, it accumulated on the surface of the nanoparticles and was recorded on the Raman spectra.

### 3.2. Magnetic Measurements

The room temperature magnetic hysteresis loops of the samples, synthesized at different reaction temperatures, are presented in Figure 3a,b, which show the size dependence of saturation magnetization and coercivity. The magnetic parameters, obtained from the curves, are summarized in Table 2, which also presents the data for magnetite nanoparticles synthesized by various methods.

As can be seen from Figure 3b, the *H_C_* curve demonstrates a growing trend and does not go through a maximum. It is known that the coercivity of nanoparticles is closely related to their size [69]. In a single domain region, the coercivity is given by

(3) HC=g−hD3/2

Refs. [70,71,72], while in the multidomain region an experimentally found equation is written approximately as

(4) $$H_{C}=a+b/D$$

Refs. [71,72], where *a*, *b*, *g*, *h* are constants, and *D* is particle size. As follows from Equations (3) and (4), coercivity should have a maximum in the size range around *D*_cr_, which corresponds to a transition from single- to multidomain states. Thus, it allows us to conclude that all synthesized samples have a single-domain structure.

The M–H curves of the samples with sizes 9 nm ≤ d_XRD_ are typical magnetization loops for superparamagnetic nanoparticles with zero or almost zero remanence magnetization and coercivity at room temperature [31,73]. However, as particle size increases, the remanence and the coercivity increase significantly, which indicates that nanoparticles with sizes d_XRD_ > 9 nm are not superparamagnetic at room temperature. The value of saturation magnetization for all samples are lower than the value M_S_ for bulk magnetite (~96 emu/g) [74]; herewith, the saturation magnetization decreases with decreasing particle size. Such behavior is attributed to the lack of full alignment of the spins even in large applied fields [75]. It is assumed that the surface of the nanoparticles consists of canted or disordered spins that prevent the core spins from aligning along the field direction, resulting in a decrease in the saturation magnetization with a decrease in particle size [39,76]. In addition, a reduced in the saturation magnetization may be due to the presence in the samples of the maghemite phase identified by Raman measurements and which has M_S_ (bulk) = 76 emu/g [77]. Another reason affecting the decrease in M_S_ is the accumulation of carbon on the surface or near-surface layer of nanoparticles during the synthesis process. Moreover, as follows from the results of Raman spectroscopy, with an increase in the reaction temperature from 350 to 380 °C, an increase in the intensity of the carbon peaks is observed, which explains a slight decrease in the saturation magnetization with an increase in particle size from 11.2 to 13.3 nm. 

As can be seen from Table 2, the values of saturation magnetization of synthesized nanoparticles are in agreement with the range of values reported in the literature for synthesis methods using iron (III) nitrate nonahydrate as a precursor. Moreover, the method demonstrates, on average, higher values compared to some other methods presented in Table 2, thus they can be used for various applications.

#### MCD Spectroscopy

**Figure 4:**
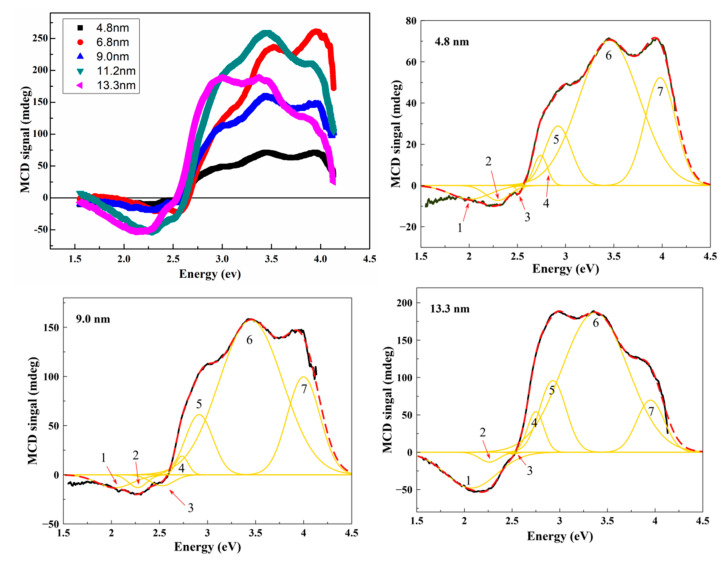
Experimental MCD spectra of the magnetite nanoparticles measured at room temperature and the best Gaussian fitting of the spectra with d = 4.8, 9, and 13.3 nm. Red dotted curves correspond the sum of the deconvoluted spectra.

The measured MCD spectra of the Fe_3_O_4_ nanoparticles with various sizes and Gaussian band fitting of the selected spectra are shown in Figure 4.

The obtained spectra have two main features: negative at energies ≤ ~2.6 eV and positive at ≥ ~2.6 eV. Besides, it can be seen that the intensity of the negative peak increases significantly when the crystallite size exceeds 9 nm. It can be explained by the loss of the superparamagnetic state by nanoparticles at room temperature, which is accompanied by an increase in magnetization. It should be noted that, although the Raman spectra revealed the presence of maghemite in the samples, two positive peaks: an intense peak at 2.8 eV and a weaker peak at around 1.8 eV [83] were not observed in our spectra. The absence of maghemite peaks in the MCD spectra indicates its low concentration in the samples and allows excluding the maghemite phase when interpreting the MCD signals. For deconvolution analysis of the MCD spectra, we have decomposed them into a set of Gaussian components. To obtain a good agreement between the experimental spectra and the sum of the components, seven Gaussian components were necessary for all samples. The fitting parameters for each component are listed in Table 3.

**Figure 5:**
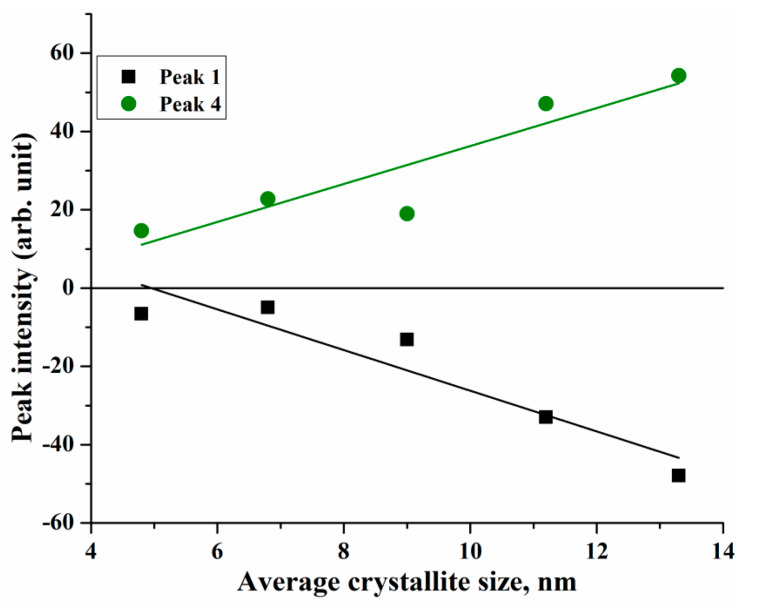
The change in the intensity of peaks 1 and 4 with increasing average crystallite size of Fe_3_O_4_ nanoparticles.

The obtained Gaussian components were identified with the electron transitions of different nature in accordance with theoretical calculations of electronic structures [84,85] and experimental studies of the magneto-optical Kerr effect [86,87] and optical magnetic circular dichroism [88] in magnetite. Based on these data from the literature, it can be concluded that the decomposition components correspond to the following transitions: peak 1 at ~2.05 corresponds to intervalence charge–transfer transition (IVCT) between two sites differing only in oxidation state, namely [Fe^2+^]_t2g_→[Fe^3+^]_eg_; peaks 2—[Fe^2+^]_t2g_→(Fe^2+^)_e_ (~2.3 eV), 4—(Fe^3+^)_t2_→[Fe^3+^]_t2g_ (~2.76 eV), and 6—[Fe^3+^]_eg_→(Fe^2+^)_t2_ (~3.44 eV) are associated with transitions between the same ions belonging to different magnetic sublattice (intersublattice charge-transfer transitions, ISCT); peaks located at ~2.53 eV (3), ~2.95 eV (5), and ~3.98 eV (7) are assigned to ligand-to-metal p—d charge transfer transitions (LMCT) across the optical gap of the spin minority involving polarized O(2p): (O2p→Fe3d). From the obtained results, it was found that the size effect is observed for peaks 1 and 4 (Figure 5) and both peaks show a growing trend of intensity with increasing crystallite size.

This behavior can be explained as follows: at low reaction temperatures, magnetite nanoparticles with smaller sizes are formed; as a consequence, they are more easily oxidized to maghemite, which is confirmed by the results of structural analysis. In this process, ${\mathrm{Fe}}_{oct}^{2+}$ ions are oxidized to Fe^3+^ in octahedral sites [89,90], which leads to the decrease in the intensity of the IVCT peak [Fe^2+^]_t2g_→[Fe^3+^]_eg_. Furthermore, it was shown in [91,92] using the Mössbauer spectroscopy that the oxidation of magnetite to maghemite is accompanied by a decrease in the fraction of trivalent iron ions in tetrahedral sites. It is assumed that this effect leads to a decrease in the intensity of the intersublattice charge-transfer transition (Fe^3+^)_t2_→[Fe^3+^]_t2g_ with the decrease in particle size.

## 4. Conclusions

A simple and inexpensive method for synthesis magnetite nanoparticles, based on thermal decomposition of iron (III) nitrate nonahydrate in HDA, is presented. Moreover, the process proceeds without the use of protective gas and at atmospheric pressure, which makes it easily reproducible. Results obtained showed that the average crystallite size increased with increasing reaction temperature. Based on the results of the analysis of the change in the lattice parameter and Raman spectroscopy, it was concluded that the samples contain a small fraction of maghemite, herewith an increase in the reaction temperature leads to the formation of more phase-pure samples. Magnetic measurements revealed that all synthesized samples are single domain, herewith when the crystallite size exceeds 9 nm, the nanoparticles cease to be superparamagnetic at room temperature. The saturation magnetization has a maximum M_S_ = 56 emu/g for the sample synthesized at T_R_ = 350 °C and the obtained values are agreement with the literature data for synthesis methods using iron (III) nitrate nonahydrate as a precursor. The MCD spectra of Fe_3_O_4_ nanoparticles were decomposed by a set of seven components that provides a good agreement between the experimental spectra and the sum of the components. The decomposition components were identified with a high degree of confidence with electronic transitions of various nature in accordance with theoretical calculations of electronic structures and experimental studies of magnetite. It was found that the intensities of the IVCT peak [Fe^2+^]_t2g_→[Fe^3+^]_eg_ and the ISCT peak (Fe^3+^)_t2_→[Fe^3+^]_t2g_ have a growing trend with increasing crystallite size, which is explained by the effect of reaction temperature on the size and phase composition of the samples.

## Figures and Tables

**Table 1:** The average crystallite size and lattice constants (a) of the samples synthesized at temperatures 200 ≤ T_R_ ≤ 380 °C.

T_R_, °C	200	260	300	350	380
d_XRD_, nm	4.8	6.8	9	11.2	13.3
a, Å	8.36	8.379	8.373	8.386	8.392

**Table 2:** Comparison of magnetic properties at room temperature and sizes of magnetite nanoparticles synthesized by various methods.

Method of Synthesis	d_XRD_, nm	Reference	M_S_, emu/g	M_R_, emu/g	H_C_, Oe
Thermal decomposition of iron (III) nitrate nonahydrate using HDA	4.8		36	0	0.6
	6.8		43	0	1
	9	This work	46	0.3	2
	11.2		56	1.5	14
	13.3		55	3.9	47
Co-precipitation	14.7	[27]	35.41	4.62	83.58
	15.1	[28]	58.722	-	27.076
	20.3	[29]	53	-	0
Hydrothermal	9.3	[30]	45	0	0
	13.4	[31]	27.2	-	58.4
	25.5	[32]	50	8	95
Sol–gel	8	[33]	47	-	0.655
	12.23	[34]	52.2	1.3	21.1
	13	[35]	35	-	17
Thermal decomposition using various reagents	5.5	[36]	43.7	-	-
	7.4	[37]	41.7	0	0
	9	[38]	65	0	1
	13.6	[39]	72	-	0
	15	[38]	70	-	12
	24.2	[40]	78.68	-	0
Various methods using iron (III) nitrate nonahydrate as a precursor	4.4 *	[78]	39.2	-	0
	6 *	[78]	52	-	0
	7 *	[79]	49	-	0
	12 *	[80]	43.6	0	0
	13	[35]	35	-	17
	~45 *	[81]	90	9	44
	~75 *	[82]	68.8	12.9	138.5

* The values obtained from TEM data.

**Table 3:** Results of the fit analysis of the MCD spectra of the Fe_3_O_4_ nanoparticles. The tetrahedral and octahedral sublattices are denoted as () and [], respectively. E—peak position (eV); I—peak intensity (arb. unit).

**Component**	**Type**	**Transition**	**d = 4.8 nm**			**d = 6.8 nm**		**d = 9 nm**		
			**E**	**I**		**E**	**I**	**E**		**I**
1	IVCT	[Fe^2+^]_t2g_→[Fe^3+^]_eg_	2.01	−6.59		2.07	−4.95	2.04		−13.15
2	ISCT	[Fe^2+^]_t2g_→(Fe^2+^)_e_	2.30	−7.23		2.34	−20.47	2.27		−12.96
3	LMCT	O(2p)→Fe(3d)	2.52	−3.4		2.56	−18.67	2.53		−11.17
4	ISCT	(Fe^3+^)_t2_→[Fe^3+^]_t2g_	2.74	14.62		2.8	22.79	2.73		19.02
5	LMCT	O(2p)→Fe(3d)	2.92	28.86		2.98	75.64	2.91		61.0
6	ISCT	[Fe^3+^]_eg_→(Fe^2+^)_t2_	3.45	70.4		3.49	230.19	3.45		157.44
7	LMCT	O(2p)→Fe(3d)	3.98	52.29		4.02	219.85	4.0		99.16
**Component**	**Type**	**Transition**	**d = 11.2 nm**				**d = 13.3 nm**			
			**E**		**I**		**E**		**I**	
1	IVCT	[Fe^2+^]_t2g_→[Fe^3+^]_eg_	2.04		−33.02		2.07		−47.91	
2	ISCT	[Fe^2+^]_t2g_→Fe^2+^)_e_	2.32		−43.28		2.26		−13.16	
3	LMCT	O(2p)→Fe(3d)	2.54		−19.63		2.51		−2.42	
4	ISCT	(Fe^3+^)_t2_→[Fe^3+^]_t2g_	2.8		47.07		2.75		54.24	
5	LMCT	O(2p)→Fe(3d)	2.99		113.51		2.93		95.95	
6	ISCT	[Fe^3+^]_eg_→(Fe^2+^)_t2_	3.45		258.06		3.37		186.92	
7	LMCT	O(2p)→Fe(3d)	3.97		148.43		3.95		69.77	
